# Molecular Characterization of Bacteria, Detection of Enterotoxin Genes, and Screening of Antibiotic Susceptibility Patterns in Traditionally Processed Meat Products of Sikkim, India

**DOI:** 10.3389/fmicb.2020.599606

**Published:** 2021-01-11

**Authors:** Meera Ongmu Bhutia, Namrata Thapa, Jyoti Prakash Tamang

**Affiliations:** ^1^DAICENTER (DBT-AIST International Centre for Translational and Environmental Research) and Bioinformatics Centre, Department of Microbiology, School of Life Sciences, Sikkim University, Gangtok, India; ^2^Biotech Hub, Department of Zoology, Nar Bahadur Bhandari Degree College, Gangtok, India

**Keywords:** meat products, 16S rRNA, ELISA, pathogens, enterotoxin, virulent genes

## Abstract

The lesser-known traditionally processed meat products such as beef *kargyong*, pork *kargyong*, *satchu*, and *khyopeh* are popular food items in the Himalayan state of Sikkim in India. The present study aimed to assess the microbiological safety of traditional meat products by identifying the potential spoilage or pathogenic bacteria, detecting the enterotoxins, and screening the antibiotic susceptibility patterns. The pH and moisture contents of the meat products varied from 5.3 to 5.9 and from 1.5 to 18%, respectively. The microbial loads of aerobic bacteria were 10^5^ to 10^7^ cfu/g, *Staphylococcus* 10^3^ to 10^6^ cfu/g, *Bacillus* 10^4^ to 10^6^ cfu/g, and total coliform 10^2^ to 10^7^ cfu/g, respectively. Based on 16S rRNA gene sequencing, the bacterial species isolated from traditionally processed meat products were *Staphylococcus piscifermentans*, *Citrobacter freundii*, *Enterococcus faecalis*, *Salmonella enterica*, *Staphylococcus aureus*, *Citrobacter werkmanii*, *Klebsiella pneumoniae*, *Macrococcus caseolyticus*, *Klebsiella aerogenes*, *Staphylococcus saprophyticus*, *Pseudocitrobacter anthropi*, *Citrobacter europaeus*, *Shigella sonnei*, *Escherichia fergusonii*, *Klebsiella grimontii*, *Burkholderia cepacia*, and *Bacillus cereus*. The enzyme-linked immunosorbent assay (ELISA) tests detected *Salmonella* spp. and enterotoxins produced by *B. cereus* well as *Staphylococcus* in a few tested samples. However, the PCR method did not detect the virulence genes of *B. cereus* and *Salmonella* in the isolates. Virulence gene (*sea*) was detected in *S. piscifermentans* BSLST44 and *S. piscifermentans* BULST54 isolated from beef *kargyong* and in *S. aureus* PSST53 isolated from pork *kargyong*. No enterotoxins were detected in *khyopeh* samples. The antibiotic sensitivity test showed that all bacterial strains were susceptible toward gentamicin, cotrimoxazole, norfloxacin, and trimethoprim. Gram-positive bacteria showed 100% sensitivity against clindamycin and erythromycin; however, 50% of the resistance pattern was observed against oxacillin followed by penicillin (33%) and ampicillin (27%).

## Introduction

Meat and meat products are important dietary cultures of many communities in the world, among which some popular meat products such as sausages, hams, salami, etc. have been extensively studied in terms of microbiological safety, health benefits, and process technology (Petit et al., 2014; Santiyanont et al., 2019; Tamang et al., 2020). Culturally and organoleptically acceptable meat products are produced by traditional methods of preservation of perishable animal flesh through fermentation (Tamang et al., 2016), sun drying (Aksoy et al., 2019), smoking (Plavsic et al., 2015), and salting (Uğuz et al., 2011). Fermented meat products are usually considered safe for consumption due to low water activity (aw) and low pH, which prevent the growth of pathogenic organisms during fermentation (Holck et al., 2017). Moreover, the presence of some dominant lactic acid bacteria in fermented meat products such as *Lactobacillus sakei*, *Lb. plantarum*, *Lb. curvatus*, *Enterococcus faecium*, *Pediococcus pentosaceus*, *Leuconostoc carnosum*, *Leuc. gelidum*, *Leuc. pseudomesenteroides*, and *Weissella* spp. (Dias et al., 2015; Laranjo et al., 2017) also inhibit the populations of pathogenic organisms in the final products. However, there is a probability of occurrence of pathogenic organisms such as Shiga toxin-producing enterohemorrhagic *Escherichia coli*, *Staphylococcus aureus*, *Salmonella*, *Campylobacter* spp., and *Listeria monocytogenes* in meat products (Morales-López et al., 2019).

Varieties of lesser-known and region-specific traditionally processed meat products, produced either by smoking/sun drying or fermentation of domesticated animals such as goat, sheep, pig, ox, and yak, are consumed by different ethnic communities in Asia. Traditionally processed meat (beef, sheep, pig, and yak) products in India are *chartayshya*, *jamma*, and *arjia* of Uttarakhand (Oki et al., 2011); *kargyong*, *satchu*, and *suka ko masu* of Sikkim; and *sa-um* of Mizoram (Rai et al., 2009; De Mandal et al., 2018). Few species of bacteria were reported earlier from *kargyong*, *satchu*, and *suka ko masu* of Sikkim, purely based on limited phenotypic and biochemical tests, which included *Lb. sakei*, *Lb. divergens*, *Lb. carnis*, *Lb. sanfransisco*, *Lb. curvatus*, *Leuc. mesenteroides*, *E. faecium*, *Bacillus subtilis*, *B. mycoides*, *B. thuringiensis*, *S. aureus*, and *Micrococcus* (Rai et al., 2010).

**FIGURE 1:**
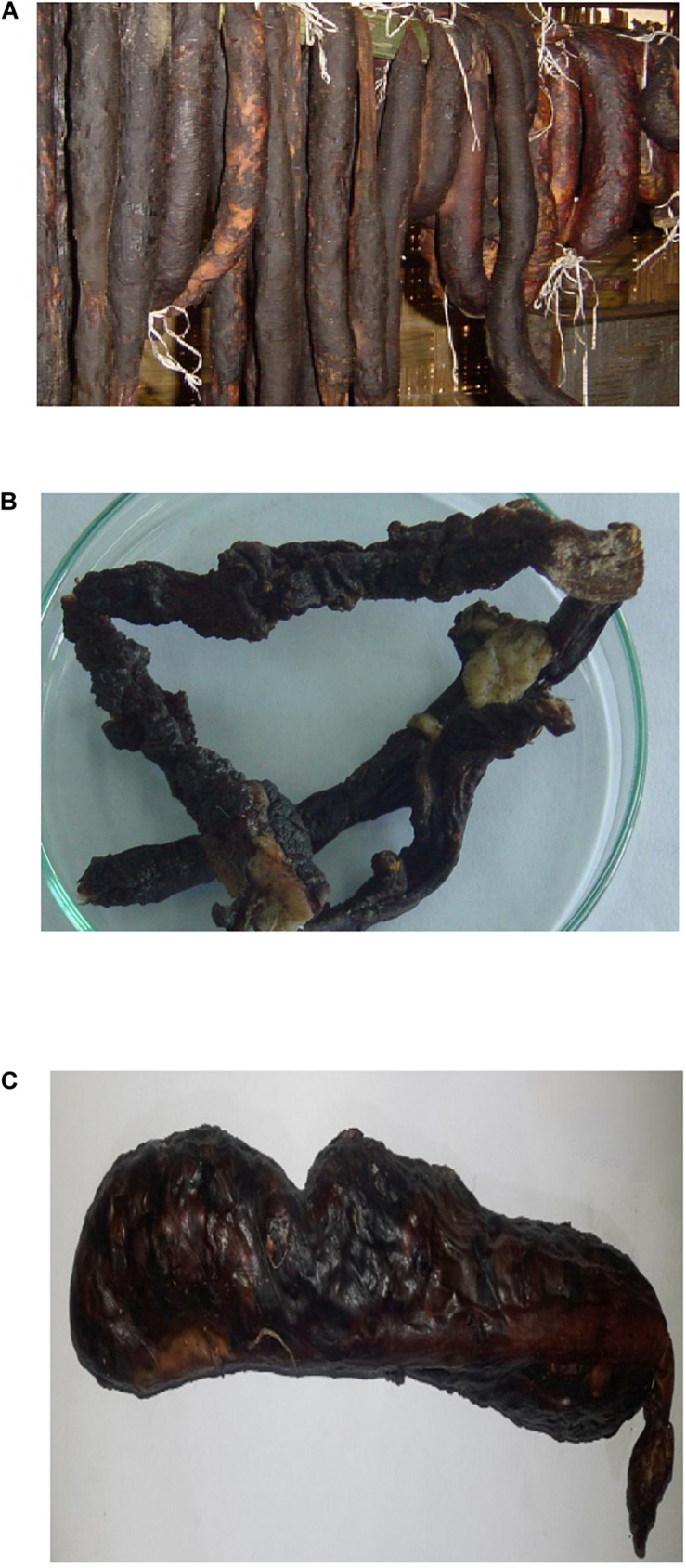
**(A)** *Kargyong* (beef and pork), **(B)** *Satchu*, and **(C)** *Khyopeh.*

In this study, four different types of traditionally processed meat products of Sikkim in India were selected viz. *kargyong* (Figure 1A), a traditional sausage-like product prepared from beef and pork meat; *satchu* (Figure 1B), a smoked beef meat product; and *khyopeh* (Figure 1C), a fermented yak meat product. *Kargyong* is produced by mixing lean meats of beef or pork with salt, garlic, and ginger, and then mixtures are stuffed into the intestine of animals as natural casings. Both ends of casings are then tied up with a rope, put into utensil, and boiled for 30 min, strained out, hooked in a bamboo stick, and smoked above an earthen oven for 10–15 days (Rai et al., 2009). No nitrates and nitrites are added during the preparation of *kargyong*. *Satchu* is prepared by slicing red meat of beef or yak into long thread-like strips; mixed with turmeric, salt, and oil; and smoked for 7–10 days (Rai et al., 2009; Rai and Tamang, 2017). *Khyopeh* is a naturally fermented yak meat product of Sikkim. During its preparation, chopped meats and innards of yak are mixed with salt, and the mixtures are stuffed into the rumen, which is previously removed from the slaughtered yak. Filled up rumen is tied up with twine and hung into bamboo stripes for natural fermentation for 4–6 months above an earthen oven (Bhutia et al., 2020). *Kargyong* and *satchu* are commonly eaten as fried side dish or curry, while *khyopeh* is eaten as soup or curry. However, the presence of pathogenic bacteria in these traditionally processed meat products has not been assessed as food safety measures. Hence, the present study is aimed to isolate and identify the pathogenic and spoilage bacteria, to detect enterotoxin genes, and also to screen antibiotic resistance patterns.

## Materials and Methods

### Sample Collection

A total of 27 samples of traditionally processed meat products viz. beef *kargyong* (eight samples), pork *kargyong* (eight), *satchu* (six), and *khyopeh* (five) were collected from different households of Sikkim in India (Table 1). Collected samples were kept in sterile polybags and put into an ice-box carrier and transported to the laboratory. Samples were stored at kept at 4°C for further analysis.

**TABLE 1:** Sequences of primers and PCR reaction conditions.

Target organism	Amplification target	Amplicon size (bp)	Sequence (5′–3′)	Reaction conditions	References
*Bacillus cereus*	*Nhea*	755	F: GTTAGGACAATCACCGC R: ACGAATGTAATTTGAGTGC	94°C, 2 min → (94°C, 60 s → 56°C, 60 s → 70°C, 120 s) 35 cycles → 72°C, 5 min	Guinebretière and Broussolle, 2002
	*Nheb*	743	F:TTTAGTAGTGGTCTGTACGC R:TTAATGTTCGTTAATCCTGC	94°C, 2 min → (94°C, 60 s → 54°C, 60 s → 72°C, 120 s) 35 cycles → 72°C, 5 min	Guinebretière and Broussolle, 2002
*Staphylococcus aureus*	*Sea*	180	F:TAAGGAGGTGGTGCCTATGG R: CATCGAAACCAGCCAAAGTT	94°C, 5 min → (94°C, 1 min → 56°C, 1 min → 68°C, 1 min) 30 cycles → 72°C, 7 min	Cremonesi et al., 2005
*Salmonella enterica*	*InvA*	275	F: TATCGCCACGTTCGGCAA R: TCGCACCGTCAAAGGAACC	95°C, 1 min → (95°C, 20 s, 55°C, 20 s → 72°C, 2 min) 35 cycles → 72°C for 4 min	Nayak et al., 2004
	*Stn*	617	F: TTGTGTCGCTATACTGGCAACC R: ATTCGTAACCCGCTCTCGTCC	94°C, 5 min → (94°C, 60 s, 59°C, 60 s → 72°C, 1 min) 35 cycles → 72°C for 10 min	Murugkar et al., 2003

### pH and Moisture

The pH was determined with a pH meter (Thermo Scientific, United States) and the moisture contents of samples were measured by a Moisture Analyzer (Ohaus MB, United States). The measurement was taken in triplicates and average values were considered.

### Food Sample Preparation

About 25 g of sample was weighed and homogenized with 225 ml buffered peptone water for 2–3 min at medium speed in a Stomacher 400 (Seward, United Kingdom) to obtain 10^–1^ dilution. Serial dilutions were made by pipetting 1 ml of homogenized samples into 9 ml of sterile diluent. Up to 10^9^ decimal dilutions were made from the previous dilution. An aliquot (0.1 ml) of the diluents was further plated on specific media by the spread plate method (Andrews and Hammack, 2003).

### Enumeration of Microbial Load

About 0.1 ml of the homogenized sample in Butterfield’s phosphate-buffered water was inoculated into Plate Count Agar (M091, HiMedia, India), Nutrient Agar (M001, HiMedia, India), Baird Parker agar (M043, HiMedia, India), *Bacillus cereus* agar (M833, HiMedia, India), and Violet Red Bile Agar (M049, HiMedia, India) using the surface spread method (Feng et al., 2002). The plates were incubated at 35°C for 24 to 48 h and the colonies were counted and results were expressed as colony-forming units per gram (cfu/g). The 3M^TM^ Petrifilm^TM^ aerobic count plate (6400, 3M, United States) was also used to enumerate the microbial load from the collected meat samples, which contained modified standard nutrients, a soluble gelling agent, and a tetrazolium indicator (Nelson et al., 2013).

### Enumeration of *Staphylococcus*

The presence or absence of *Staphylococcus* in each sample was assessed according to the Food and Drug Administration (FDA) standard method (Tallent et al., 2016). Baird–Parker agar plates were supplemented with Egg Yolk Tellurite Emulsion (FD046L, HiMedia, India). Plates were incubated at 37°C for 36–48 h. Convex, black, shiny colonies with narrow white margin surrounded by a clear zone were regarded as *Staphylococcus* (Tallent et al., 2016).

### Enumeration of *Enterococcus*

Ten grams of each sample was diluted in 90 ml of peptone water (0.1% w/v) for homogenization by mechanic stirring. In products with high-fat content, 1% v/v Tween 80 (P1754, Merck, Germany) was added. Then, 0.1 ml aliquots of diluents from homogenized food were spread in Bile Esculin Azide Agar (M493, HiMedia, India) plates and incubated for 24 h at 35°C. Colonies that showed black pigmentation on the BEA agar were regarded as *Enterococcus* (Delpech et al., 2012).

### Enumeration of *Bacillus*

Ten grams of each sample was blended in 90 ml of 0.85% sterile saline in using Stomacher 400 (Seward, United Kingdom) for 3 min followed by serial dilution. Aliquots of 0.1 ml of the appropriate dilutions were surface plated on prepoured plates of *B. cereus* agar (M833, HiMedia, India). Blue colonies with positive precipitation were regarded as *Bacillus* (Rai et al., 2010).

### Enumeration of *Escherichia* and Coliform Bacteria

Ten grams of each sample was homogenized in a Stomacher 400 (Seward, United Kingdom) for 3 min in 90 ml buffered peptone water and serial dilution was prepared. An aliquot (0.1 ml) of the homogenate was transferred to Violet Red Bile Agar (M049, HiMedia, India) plates and incubated at 18–24 h at 35°C. Colonies that are purple red, 0.5 mm or larger, and surrounded by a zone of precipitated bile acids were regarded as coliforms. The colonies were further transferred to Brilliant Green Bile Broth (M1211, HiMedia, India) and incubated at 35°C for 24–48 h for gas production. Pure culture of isolates was inoculated in Eosin Methyl Blue Agar (M317, HiMedia, India) and MacConkey Agar (M0081B, HiMedia, India) for differentiation of enteric bacteria (Feng et al., 2002).

### Enumeration of *Salmonella*

The method for isolation of *Salmonella* was followed as per the protocol described by Santos et al. (2020). About 25 g of the sample was aseptically homogenized and inoculated into 225 ml of buffered peptone water (M614, HiMedia, India) in a sterile jar. The pre-enrichment culture was incubated at 37°C for 18–24 h. Then, 0.1 ml of pre-enrichment culture was inoculated into 10 ml Rappaport–Vassiliadis (RV) medium (M880, HiMedia, India) and incubated at 42°C for 24 h in a circulating, thermostatically controlled water bath (1322, Remi, India). A loopful of cultured broth was streaked onto Xylose-Lysine Deoxycholate Agar (M031, HiMedia, India) plates and incubated at 37°C for 18–24 h. Pink colonies with or without black color were regarded as *Salmonella*. Each isolate was streaked onto Nutrient Agar (M001, HiMedia, India), incubated at 37°C for 24 h, and phenotypically characterized.

### Phenotypic Characterization

The isolated bacteria were characterized on the basis of colony morphology (Tallent et al., 2016), Gram’s reaction, sporulation test (Hussey and Zayaitz, 2007), motility tests (Shields and Cathcart, 2011), enzymatic reactions, and biochemical tests.

### Enzymatic Tests

Catalase test was performed by placing a loopful of bacterial isolates into the test tube containing five to six drops of 3% hydrogen peroxide. A positive reaction was represented by the presence of bubbles and no bubble formation represented catalase negative (Reiner, 2010). For the urease test, a heavy inoculum from 24 h pure culture was streaked into the entire slant surface of a tube containing Christensen’s medium and incubated at 35°C. The slant was observed for a color change up to 6 days. Urease production was indicated by a bright pink color on the slant that extends into the butt (Brink, 2010). A gelatin hydrolysis test was performed by stab-inoculating a 24-h culture into the tubes containing nutrient gelatin. The inoculated tubes and uninoculated control tubes were incubated at 25°C for up to 1 week and checked every day for gelatin liquefaction. Liquefaction due to gelatinase activity was confirmed by immersing tubes in an ice bath for 15–30 min, and then tubes were tilted to observe if the gelatin was hydrolyzed. The positive test of hydrolyzed gelatin resulted in a liquid medium after exposure to cold temperature, whereas the negative tests remained solid (dela Cruz and Torres, 2012). For the nitrate reduction test, bacterial cultures were grown in 5 ml nitrate broth for 12–24 h at 35°C. One milliliter of the culture was mixed with three drops of reagents (reagent A and reagent B) and observed for the development of a red/yellow color, indicating the presence/absence of nitrate. A small amount of zinc dust was added to the tube for 5 days to observe for the development of a red color, indicating the absence of nitrate reduction (Buxton, 2011). Coagulase and DNA hydrolysis tests were specifically performed for the characterization of *Staphylococcus*. For the coagulase test, about three to four isolated colonies of suspected *Staphylococcus* were emulsified in 0.3 ml of Brain Heart Infusion Broth (LQ210D, HiMedia, India) and incubated at 35–37°C for 18–24 h. Then, 0.5 ml of reconstituted plasma was added into the BHI culture, mixed thoroughly, and incubated at 37°C. The culture was observed for clot formation at intervals for over the next 46 h. The positive cultures exhibited clotting by the end of 24 h and negative cultures showed the absence of clot formation (Bennett et al., 1986). For the DNA hydrolysis test, pure culture of suspected *Staphylococcus* was streaked on DNase Test Agar w/Methyl Green (M1419, HiMedia, India) and incubated at 37°C for 24 h. A positive result was indicated by a clear halo around the growth (Kateete et al., 2010).

### Biochemical Tests

#### Carbohydrate Fermentation

Aseptically, two to three drops of the test organism from 18 to 24 h BHI broth culture was inoculated in a Phenol Red Carbohydrate Broth containing Durham’s tube and incubated at 37°C for 24 h. A yellow color indicated fermentation of the sugar with lowering of pH to 6.8 or less. A delayed fermentation produces an orange color and bubble trapped within the Durham tube indicated gas production. A reddish or pink color indicated a negative reaction (Reiner, 2012).

#### IMVic (Indole, Methyl Red, Voges–Proskauer, and Citrate Utilization) and Indole Tests

Pure culture of an isolate was inoculated in the tube of Tryptone Broth (M463, HiMedia, India) and incubated at 37°C for 24–48 h. Five drops of Kovac’s indole reagent (R008, HiMedia, India) was added directly onto the cultured tube. A positive indole test was indicated by the formation of pink to red color (cherry red ring) in the reagent layer on top of the medium. A negative test was indicated by yellow color on top of the reagent layer (MacWilliams, 2012).

#### Methyl Red and Voges–Proskauer Test

The fresh tested culture was inoculated in 5 ml Methyl Red–Voges–Proskauer (MR-VP) broth (LQ082, HiMedia, India) and incubated for 48 h at 35°C. Then, 2.5 ml of culture was transferred into a new sterile tube and five drops of Methyl Red (MR) Reagent (I007, HiMedia, India) was added. The MR-positive test organism showed red coloration of the medium and the negative test organisms showed yellow coloration of the medium due to low acid production (McDevitt, 2009). In the remaining culture grown in MR-VP broth, 0.6 ml of Barritt’s reagent A (R029, HiMedia, India) and 0.2 ml of Barritt’s reagent B (R030, HiMedia, India) were added. The tubes were then shaken for 30 s to 1 min and the tubes were allowed to stand for 30 min to 1 h. The VP-positive organism showed red coloration on top of the culture and the VP-negative organism showed yellowish color (McDevitt, 2009).

#### Citrate Test

A Simmon citrate medium (M099, HiMedia, India) was prepared in a tube slant and the fresh pure test organism was inoculated on the surface of the slant, incubated at 37°C for 18–24 h. A positive organism was represented by growth on the surface and change in color from the original green to blue (MacWilliams, 2009).

### Genomic Characterization

#### DNA Extraction

DNA was extracted from each bacterial isolate by the standard phenol/chloroform method of Cheng and Jiang (2006). The quality of DNA was checked by electrophoresis in 0.8% agarose gel and quantified using Nano-Drop ND-1000 spectrometer (Eppendorf, Germany).

#### PCR Amplification

The PCR of 16S rRNA gene from isolated DNA was amplified using a universal oligonucleotide primer pair 27F (5′-AGAGTTTGATCCTGGCTCAG-3′) and 1492R (5′-TACGGTTACCTTGTTACGACTT-3′) (Lane, 1991) in a thermal cycler (Thermo Fisher, United States). The reaction mixture, conditions, and protocol for the polymerase chain reaction amplification were done following the method of Chagnaud et al. (2001). The PCR amplification was performed in a mixture containing a final volume of 50 μl of GoTaq Green Master Mix (2×) (M7122, Promega, United States), 10 μM of F primer, 10 μM of R primer, and nuclease-free water (NEB). The PCR reaction program was set under the following PCR conditions: 94°C for 10 min; 94°C for 1 min, 65°C for 1 min, and 72°C for 30 s for 35 cycles; and 72°C for 7 min. The PCR products were detected by electrophoresis using 1% agarose, and the bands were stained with 7 μl/100 ml of ethidium bromide and visualized using Gel Doc EZ Imager (Bio-Rad, United States). A standard 100-base pair DNA ladder was used for the verification of amplicon size. The amplified PCR products were purified using the PEG (polyethylene glycol)–NaCl (sodium chloride) (20% w/v of PEG, 2.5 M NaCl) precipitation method of Schmitz and Riesner (2006).

### 16S rRNA Gene Sequencing

The PCR products were set up in 5 μl volume for a single primer amplification with the same universal primers 27F (5′-AGAGTTTGATCCTGGCTCAG-3′) and 1492R (5′-TACGGTTACCTTGTTACGACTT-3′) (Lane, 1991) for separate reactions of each primer. The PCR reaction was set as follows: denaturation (96°C, 10 s), annealing (50°C, 5 s), and elongation (60°C, 2 min) with a stop reaction at 4°C. The amplicons were then precipitated with 1 μl sodium acetate (3 M, pH 5.2) and 24 μl of absolute alcohol, mixed briefly in vortex, and incubated at room temperature for 15 min, centrifuged at 12,000 rpm for 20 min, further washed with 70% ethanol, air-dried, and suspended in 10 μl formamide. Sequencing of the amplicons was performed by the Sanger sequencing method of Heather and Chain (2016) which was carried out in an automated DNA Analyzer (ABI 3730XL Capillary Sequencers, Applied Biosystems, Foster City, CA, United States).

#### Data Analysis

The quality of sequences was analyzed by Sequence Scanner v.1.0 (Applied Biosystems, Foster City, CA, United States) followed by DNA sequence assembly using ChromasPro 2.1.8^1^. The contigs were subjected to BLAST (Basic Local Alignment Search Tool) (Benson et al., 2014) for nucleotide similarity search. The sequences were then aligned by pairwise alignment using ClustalW and the phylogenetic tree was constructed by the neighbor-joining method using MEGA7.0 software (Kumar et al., 2016). The genera and species were identified based on the lowest *E*-value in BLAST.

### Detection of Enterotoxin by ELISA

Samples were analyzed for detection of staphylococcal enterotoxin, *Bacillus* diarrheal enterotoxin, and *Salmonella*. For the extraction of *Bacillus* diarrheal enterotoxin, 25 g of the sample was added into 50 ml of 0.25 M Tris buffer (pH 8) and blended for 3 min. The slurry was transferred to a centrifuge bottle and centrifuged for 10 min at 5,000 rpm. The supernatant was then filtered through a prepared syringe and the eluate was adjusted to pH 7.0–8.0. Then, 5 ml of the eluate was mixed thoroughly with 50 μl of the sample additive (Rahmati and Labbe, 2008). The sample was then subjected to an enzyme-linked immunosorbent assay (ELISA) test using Tecra *Bacillus* diarrheal enterotoxin visual immunoassay (BDEVIA48, 3 M, United States) according to the manufacturer’s instructions. For the extraction of staphylococcal enterotoxin, 10 g of the sample was added into 15 ml PBS (pH 7.4) and homogenized for about 3 min in a blender. The test sample was centrifuged at 5,000 rpm for 10 min at 10°C and 100 μl of the supernatant was tested with a RIDASCREEN staphylococcal enterotoxin assay (SET Total) kit (R4105, R-Biopharm, Germany) for bulk detection of the enterotoxins *SEA*, *SEB*, *SECl*, *SEC2*, *SEC3*, *SED*, and *SEE* (Bennett, 2005). The test was performed according to the manufacturer’s instructions. For detection of *Salmonella* spp., 25 g of the sample was mixed with 225 ml of lactose broth and incubated at 36°C (±1°C) for 22–26 h. Then, 0.1 ml of the primary enriched sample was transferred into 10 mL of Rappaport–Vassiliadis broth and further incubated at 42°C (±1°C) for 18–24 h. One milliliter of the enrichment sample was transferred into 25 μl of sample additive in a tube and heated for 15 min in a water bath and then subjected to ELISA test using the Tecra *Salmonella* Visual immunoassay (SALVIA96, 3 M, United States) according to the manufacturer’s instructions (Perez-Montano et al., 2012). Although all positive results were visible with the naked eye, a microplate reader and PC software were also used to measure the results. The absorbance of the samples was measured by determining optical densities at 450 nm using the iMark Microplate Reader (Bio-Rad, United States). The positive control had an absorbance of ≥1.0 and the negative control had an absorbance of ≤0.2 as per the manufacturer’s instruction.

### Detection of Enterotoxin Genes by PCR

The identified bacterial isolates were tested for the detection of few enterotoxin genes viz. *Staphylococcus* (*sea*), *B. cereus* (*nhea*, *nheb*), and *Salmonella* (*invA*, *stn*) by PCR analysis. These enterotoxin genes were selected based on their prevalence associated with foodborne illness (Kérouanton et al., 2007; Wallin-Carlquist et al., 2010; Tekale et al., 2015; Amor et al., 2019). The PCR amplification was performed in a total volume of 25 μl and the reaction mixture contained GoTaq Green Master Mix (Taq DNA polymerase, dNTPs, MgCl_2_, and reaction buffers), 10 μM of each primer [Imperial Life Sciences (P) Ltd, India], and nuclease-free water (P1193, Promega, United States). The amplification target genes, amplicon size in bp, primer sequences, and reaction conditions used in this experiment are given in Table 1. Ten-microliter aliquot of the PCR product was resolved on 2% agarose gel at 80 V and documented with a camera system (Bio-Rad, United States). For the positive control, a reference strain of *Salmonella enterica* ser. Typhimurium (MTCC 3223), *B. cereus* (MTCC 1272), and *S. aureus* (MTCC 740) obtained from IMTECH, India, was used. *Bacillus nitratireducens* (MK203014), *Enterobacter hormaechei* (MK748263), and *Staphylococcus warneri* (MK203007) were used as a negative control for the test.

### Antibiotic Sensitivity Test

Nineteen bacterial isolates were evaluated for antimicrobial sensitivity test using the Kirby–Bauer disk diffusion method according to Clinical and Laboratory Standard Institute (CLSI) guidelines (Patel et al., 2017). The inoculum suspension was prepared by selecting four to five pure isolated colonies from overnight growth (16–24 h of incubation) on a non-selective medium with a cotton swab and suspending the colonies into sterile 0.85% physiological saline water. The density of the suspension was compared to a 0.5 McFarland Standard (R092, HiMedia, India), and turbidity was adjusted to McFarland 0.5 by adding saline or more organisms. Another sterile cotton swab was dipped into an inoculum suspension and the excess fluid was removed to avoid overinoculation of plates by pressing against the wall of the inside of the tube above the fluid level. The inoculum was spread evenly over the entire surface of Mueller–Hinton Agar (M173, HiMedia, India) plates by swabbing in three directions and allowed to stand for 3 to 5 min for drying. The standard antibiotic disks (HiMedia, India) containing amoxicillin/clavulanate (30 μg), ceftazidime (30 μg), cefuroxime (30 μg), cefepime (30 μg), trimethoprim (5 μg), nitrofurantoin (300 μg), nalidixic acid (30 μg), tobramycin (10 μg), ciprofloxacin (5 μg), erythromycin (15 μg), chloramphenicol (30 μg), penicillin G (10 μg), ampicillin (10 μg), streptomycin (10 μg), tetracycline (30 μg), gentamicin (10 μg), vancomycin (30 μg), oxacillin (1 μg), rifampicin (5 μg), cefoxitin (30 μg), aztreonam (30 μg), clindamycin (2 μg), cotrimoxazole (25 μg), norfloxacin (10 μg), cefotaxime/clavulanic acid (30 μg), and ceftriaxone (30 μg) were dispensed onto inoculated MHA plates using the disk dispenser. The antibiotic disks used were based on their availability at the laboratory at the time of study. The plates were allowed to stand for a few minutes and were incubated for 24 h at 37°C. Antibiotic sensitivity was checked by measuring the zone of inhibition from the back of the plate to the nearest millimeter using a ruler. The zone of inhibition was recorded and compared with the zone diameter interpretative chart to determine whether the bacterial isolates were susceptible, intermediate, or resistant by referring to the CLSI Performance Standards for Antimicrobial Susceptibility Testing (Patel et al., 2017) and journals (Gao et al., 2018; Thung et al., 2018). The bacteria were reported as sensitive (S), intermediate (I), or resistant (R) to each of the antibiotics used in the test. The positive control strains used for the test includes *Escherichia coli* (MTCC 443, IMTECH, India) for Gram-negative bacteria and *S. aureus* (MTCC 96, IMTECH, India) for Gram-positive bacteria. Sterile water was used as negative control.

## Results

### Microbial Population

A high pH was recorded in *khyopeh* with 5.9 and a lower pH in *satchu* with a mean pH of 5.5, indicating a slightly acidic nature of the meat samples (Table 2). We observed a very low percentage of moisture content in *khyopeh* with an average value of 2.5% (1.5–3.5%), and a higher moisture content was observed in beef *kargyong* with a mean value of 14.1%. A higher mean value of aerobic bacterial count was observed in beef *kargyong* with 9.3 × 10^6^ cfu/g and a lower mean value of 1.2 × 10^6^ cfu/g was found in *khyopeh*, respectively. Staphylococcal count was found highest in *satchu* with an average value of 1.8 × 10^6^ cfu/g and least in *khyopeh* with a mean value of 1.0 × 10^3^ cfu/g, whereas *Bacillus* count was found highest in beef *kargyong* with a mean value 4.1 × 10^5^ cfu/g and lowest in *satchu* (2.2 × 10^5^ cfu/g). Interestingly, growth of *Bacillus* was not observed in *khyopeh.* Total coliform count was found highest in *satchu* with a mean value of 1.2 × 10^7^ cfu/g and lowest in *khyopeh* (2.1 × 10^2^ cfu/g) (Table 2).

**TABLE 2:** Collection sites, altitude, moisture, pH, and bacterial load of traditionally processed meat products of Sikkim.

Sample	District (number of samples)	Collection site	Altitude (m)	Moisture content (%)	pH	Bacterial load (cfu/g)			
						Aerobic bacteria (10^6^)	*Staphylococcus* (10^5^)	*Bacillus* (10^5^)	Total coliform (10^6^)
Beef *kargyong*	East Sikkim (4)	Lalbazaar, Gangtok	1,639	18.0 (16.3–19.7)	5.8 (5.5–6.1)	24.0 (23.0–25.0)	25.0 (23–27)	2.2 (2.1–2.3)	0.82 (0.25–1.4)
		Martam	1,652	15.1 (14.7–15.5)	5.8 (5.8–5.9)	12.3 (2.4–13)	2.0 (1.8–2.2)	1.9 (1.8–2.1)	1.4 (0.21–2.6)
	West Sikkim (2)	Geyzing	1,443	12.8 (12.4–13.3)	5.9 (5.8–6.1)	0.22 (0.18–0.27)	2.0 (2.0–2.1)	2.1 (1.6–2.7)	0.27 (0.26–0.29)
	South Sikkim (2)	Kitam	535	10.8 (10.2–11.4)	5.7 (5.6–5.8)	1.0 (0.23–1.9)	1.9 (1.7–2.1)	10.5 (2.1–19)	0.9 (0.21–1.7)
Pork *kargyong*	East Sikkim (4)	Lalbazaar, Gangtok	1,639	14.8 (8.6–16.5)	5.3 (5.2–5.4)	0.74 (0.28–1.2)	23.0 (22.0–24.0)	11.5 (2.0–21)	0.22 (0.21–2.3)
		Baluakhani Rd	1,722	8.8 (8.6–9.1)	5.4 (5.4–5.5)	0.22 (0.21–0.23)	15.0 (15.0–16.0)	2.4 (2.3–2.6)	2.2 (2.1–2.3)
	West Sikkim (2)	Geyzing	1,443	11.8 (11.3–12.4)	5.8 (5.8–5.9)	1.2 (0.1–2.2)	0.74 (0.18–1.3)	1 (0.2–1.8)	0.15 (0.13–0.18)
	South Sikkim (2)	Kitam	535	9.4 (9.1–9.7)	5.8 (5.8–5.9)	1.8 (1.7–1.9)	2.2 (1.9–2.6)	1.2 (0.15–2.3)	1.0 (0.2–1.8)
*Satchu*	East Sikkim (4)	Lalbazaar, Gangtok	1,639	7.3 (7.1–7.6)	5.3 (5.3–5.4)	1.2 (0.21–2.2)	10.9 (1.8–20)	1.7 (1.5–1.9)	34.0 (23–45)
		Martam	1,652	7.6 (7.2–8.1)	5.3 (5.3–5.4)	2.8 (2.7–2.9)	19.5 (16.0–23.0)	4.6 (4.5–4.7)	1.5 (0.27–2.8)
	West Sikkim (2)	Geyzing	1,443	10.7 (6.8–7.1)	5.7 (5.7–5.8)	1.4 (1.3–1.6)	2.2 (2.1–2.3)	0.38 (0.17–0.21)	0.16 (0.12–0.20)
*Khyopeh*	North Sikkim (5)	Lachung	2,625	2.5 (1.5–3.5)	5.9 (5.8–6.1)	1.2 (0.15–2.3)	0.01 (0.002–0.02)	Absent	0.00021 (0.00012–0.00031)

**cfu/g, colony-forming units per gram*.*

### Identification

Selective media were used to isolate some foodborne bacterial pathogens and spoilage bacteria. A total of 128 bacteria were isolated from traditional meat samples. On the basis of cultural characteristics, cell morphology, and carbohydrate fermentation tests, bacterial genera were preliminarily identified using a taxonomical key (Holt et al., 1994) as *Enterobacter*, *Klebsiella*, *Escherichia*, *Salmonella*, *Enterococcus*, *Bacillus*, *Staphylococcus*, *Citrobacter*, and *Pseudomonas* (Table 3). Out of the 128 bacterial isolates, 19 representative isolates were selected from each grouped strain having similar phenotypic characteristics (data not shown) for molecular identification.

**TABLE 3:** Phenotypic and biochemical characterizations of bacteria isolated from traditionally processed meat products of Sikkim.

Colony morphology				Enzymatic reactions										Sugar fermentation									IMViC test					
					
	Media	Gram reaction	Motility	Catalase	Coagulase reaction	Urease reaction	DNase	Nitrate reduction	Gelatin hydrolysis	Lactose	Sorbitol	Arabinose	Glucose	Mannitol	Mannose	Trehalose	Raffinose	Maltose	Xylose	Rhamnose	Sucrose	Ribose	Adonitol	Indole	Methyl red	Voges–Proskauer	Citrate	Tentative identification (total number of isolates)
Pink without sheen	EMB	−	+	+	−	−(6) v(4)	−	+	−	+(5) v(5)	+	+	+	+	+	+	+(4) −(6)	+	+	+	+	+	−(7) v(3)	−(8) v(2)	−	+	+(6) v(4)	*Enterobacter* (10)
Pink, mucoidy	EMB	−	−	+	−	+	+	+	+	+(8) −(7)	+	+	+	+	+	+	+	+	+	+(10) v(5)	−(6) v(9)	+	+	−	+(12) −(3)	+(13) −(2)	+(11) −(4)	*Klebsiella* (15)
Purple with metallic sheen	EMB	−	+	+	−	−	−	+	−	+(10) v(2)	+(10) −(2)	+	+(8) v(4)	+(9) −(3)	−	+	+	−	+	+(9) v(3)	−(8) v(4)	−	−	+(7) v(5)	+	−	−(5) v(7)	*Escherichia* (12)
Red colonies with black centers	XLD	−	+	+	−	−	−	+	−	−(5) v(3)	+	+	+	+	+ −	+	−	+	+	+(4) −(4)	−(3) +(5)	−	−	−	+	−	+(3) −(5)	*Salmonella* (8)
Blackening of media	BEA	+	−	−	−	−	−	+	−	+	+	−	+	+	+	+	−	+	−	−(10) v(6)	+	+	+	−	−	+	−	*Enterococcus* (16)
Blue	BCA	+	+	+	−	+	−	v(3) +(9)	−	−	−	−(3) +(9)	−	+	−	+	+ −	+	−	−	+	+	−	−	−	−(3) +(9)	+	*Bacillus* (12)
Gray-black	BPA	+	+	+	+(15) −(5)	+(12) −(8)	+	+(9) −(11)	+	+	+(3) v(17)	−(12) v(8)	+	+	+	+	−(19) v(1)	+	−	+	+	+	−	−	−	+(11) −(5) v(4)	–	*Staphylococcus* (20)
Purple-blue black	EMB	−	−	+	−	v	−	+	−	v	+	+	+	−	+	+	−	v	+	+	−	−	+(17) −(8)	+(16) −(9)	+	−	+	*Citrobacter* (25)
Pale-green	NA	−	+	+	−	−(8) +(2)	−	+(7) −(3)	+	−	−	+(8) −(2)	+	+(9) −(1)	−	−	−	−	−	+(3) −(7)	−	−	−	−	−	−	+	*Pseudomonas* (10)

**IMViC test, indole, methyl red, Voges–Proskauer, and citrate tests; EMB, eosin methylene blue; XLD, xylose lysine deoxycholate; BEA, bile esculin agar; BCA, Bacillus cereus agar; NA, nutrient agar; −, negative; +, positive; v, variable; number in parenthesis indicates number of isolates*.*

**FIGURE 2:**
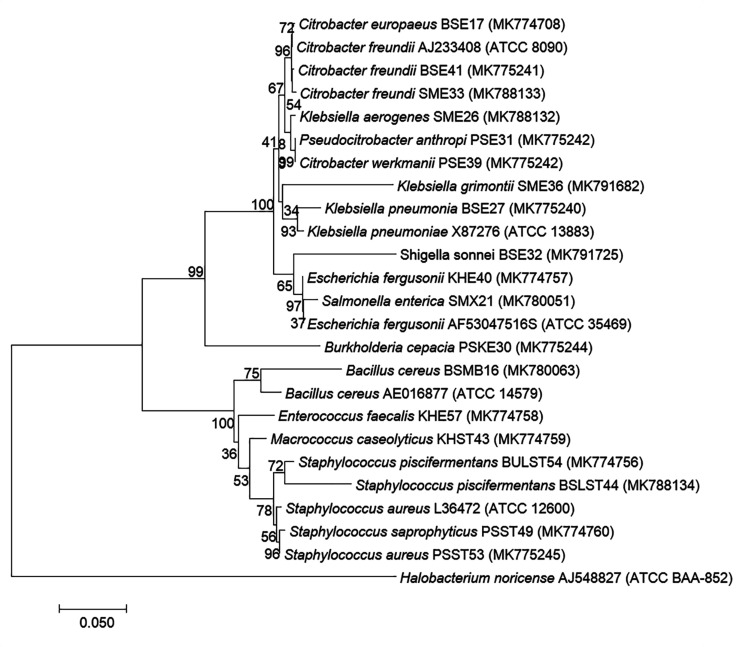
Molecular phylogenetic analysis of 19 bacterial isolates recovered from traditional processed meat products based on 16S rRNA region sequencing. Neighbor-joining phylogenetic tree representation by MEGA 7 with *Halobacterium noricence* AJ548827 as the outgroup with the evolutionary history (Kumar et al., 2016). The optimal tree with a sum of branch length = 1.13658194 and the percentage of replicate trees in which the clustered associated taxa in the bootstrap test (1,000 replicates) are shown next to the branches. The trees are drawn to scale, with branch lengths in the same units as those of the evolutionary distances used to infer the phylogenetic tree (Tamura et al., 2004), and are expressed in units of the number of base substitutions per site. Analysis involved 25 nucleotide sequences with a total of 569 positions in the final dataset.

**FIGURE 3:**
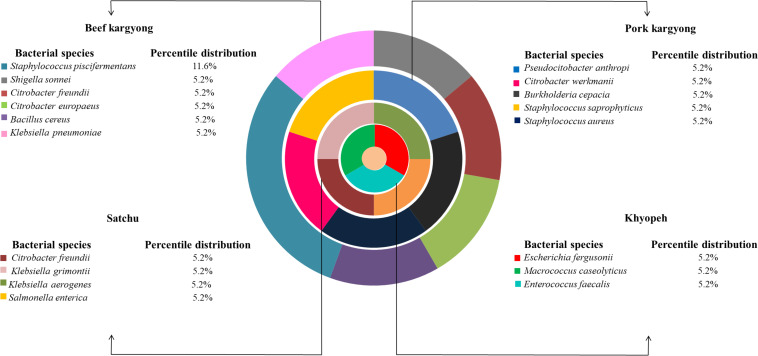
Composition of bacterial species in various meat products of Sikkim.

Based on 16S rRNA gene sequencing (Figure 2 and Table 4), bacterial species were taxonomically confirmed as *Staphylococcus piscifermentans*, *Citrobacter freundii*, *S. aureus*, *Enterococcus faecalis*, *S. enterica*, *Citrobacter* werkmanii, *Klebsiella pneumoniae*, *Macrococcus caseolyticus*, *Klebsiella aerogenes*, *Staphylococcus saprophyticus*, *Pseudocitrobacter anthropi*, *Citrobacter europaeus*, *Shigella sonnei*, *Escherichia fergusonii*, *Klebsiella grimontii*, *Burkholderia cepacia*, and *B. cereus*. The percentile compositions of bacterial species in various meat products of Sikkim are shown in Figure 3.

**TABLE 4:** Identification of bacterial strains isolated from traditionally processed meat products of Sikkim by 16S rRNA gene sequence based on the Basic Local Alignment Search Tool (BLAST).

Product	Isolate code	Identity	Type species (% similarity)	GenBank accession number	Size (base pair)
Beef *kargyong*	BSE32	*Shigella sonnei*	*Shigella sonnei* CECT 4887 (93.09%)	MK791725	1,073
	BSE27	*Klebsiella pneumonia*	*Klebsiella pneumoniae* subsp. *rhinoscleromatis* R-70s (98.55%)	MK775240	1,442
	BSE41	*Citrobacter freundii*	*Citrobacter freundii* ATCC 8090 (99.16%)	MK775241	1,420
	BSE17	*Citrobacter europaeus*	*Citrobacter europaeus* 97/79 (97.23%)	MK774708	1,037
	BSLST44	*Staphylococcus piscifermentans*	*Staphylococcus piscifermentans* CIP103958 (98%)	MK788134	1,124
	BULST54	*Staphylococcus piscifermentans*	*Staphylococcus piscifermentans* CIP103958 (97.89%)	MK774756	1,088
	BSMB16	*Bacillus cereus*	*Bacillus cereus* ATCC 14579 (97%)	MK780063	1,040
Pork *kargyong*	PSE31	*Pseudocitrobacter anthropi*	*Pseudocitrobacter anthropi* C138 (98.24%)	MK775242	1,420
	PSE39	*Citrobacter werkmanii*	*Citrobacter werkmanii* CDC 0876-58 (97.96%)	MK775243	1,415
	PSKE30	*Burkholderia cepacia*	*Burkholderia cepacia* ATCC 25416 (98.36%)	MK775244	1,460
	PSST49	*Staphylococcus saprophyticus*	*Staphylococcus saprophyticus* subsp. *saprophyticus* ATCC 15305 (98.81%)	MK774760	1,424
	PSST53	*Staphylococcus aureus*	*Staphylococcus aureus* ATCC 12600	MK775245	1,433
*Satchu*	SME36	*Klebsiella grimontii*	*Klebsiella grimontii* SB73 (92.47%)	MK791682	1,126
	SME26	*Klebsiella aerogenes*	*Klebsiella aerogenes* NCTC10006 (98.28%)	MK788132	1,418
	SMX21	*Salmonella enterica*	*Salmonella enterica* subsp. *arizonae* DSM 9386 (97%)	MK780051	1,432
	SME33	*Citrobacter freundii*	*Citrobacter freundii* ATCC 8090	MK788133	1,423
*Khyopeh*	KHE40	*Escherichia fergusonii*	*Escherichia fergusonii* ATCC 35469 (99.58%)	MK774757	1,415
	KHST43	*Macrococcus caseolyticus*	*Macrococcus caseolyticus* subsp. *hominis* CCM 7927 (99.16%)	MK774759	1,434
	KHE57	*Enterococcus faecalis*	*Enterococcus faecalis* ATCC 19433 (98.26%)	MK774758	1,433

**ATCC, American type culture collection; DSM, Deutsche Sammlung von Mikroorganismen; CCM, Czech Collection of Microorganisms; CECT, Colección Española de Cultivos Tipo; CDC, Centers for Disease Control and Prevention*.*

### Occurrence of Bacterial Enterotoxins

**FIGURE 4:**
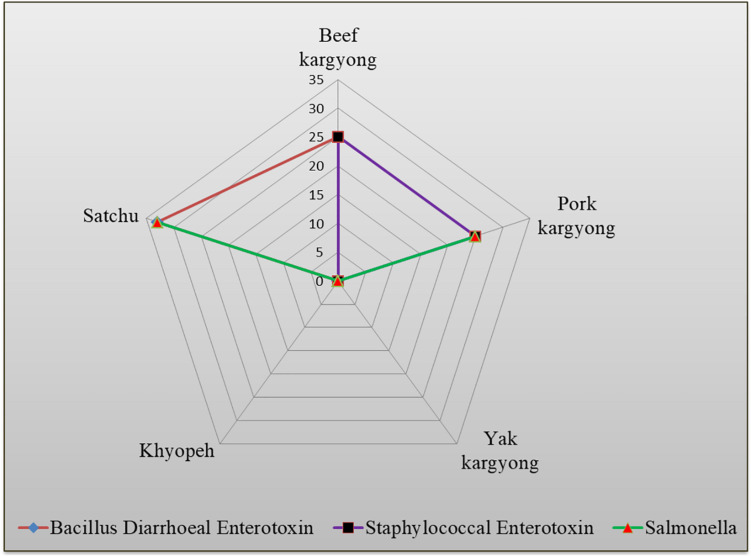
Percentile distribution of different bacterial enterotoxins present in traditional meat products.

**FIGURE 5:**
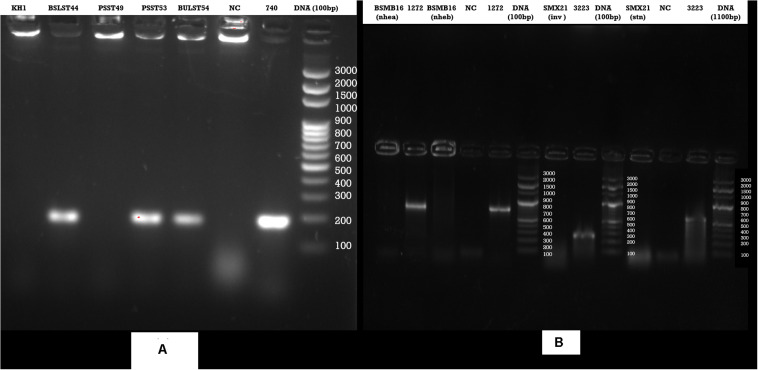
Agarose gel analysis of target strains after PCR amplification of DNA **(A)** *Staphylococcus* enterotoxin gene (*sea*), **(B)** *Bacillus cereus* enterotoxin genes (*nhea, nheab*) and *Salmonella* (*inv, stn*) enterotoxin genes isolated from traditional meat products.

Enzyme-linked immunosorbent assay tests were performed on 27 samples of meat products, out of which two samples of beef *kargyong* tested positive for *Bacillus* diarrheal enterotoxins and staphylococcal enterotoxins (Table 5), two samples of pork *kargyong* tested positive for *Salmonella* spp. as well staphylococcal enterotoxins, and two samples of *satchu* tested positive for *Bacillus* diarrheal enterotoxins and *Salmonella* spp. However, all the tested enterotoxins were absent in the *khyopeh* sample. The overall prevalence of enterotoxins in the meat products detected by ELISA test is shown in Figure 4. The optical densities for each extracted sample were also measured at 450 nm with a microtiter reader. The minimum amount of detection limit (LOD) is 0.2 ng/ml of the sample according to the manufacturer’s recommendations. We found varied detectable limits with extracts of beef/pork/yak *kargyong*, *satchu*, and *khyopeh* ranging from 0.08 ng/ml in yak *kargyong* to 0.35 ng/ml in beef *kargyong* for *Bacillus* diarrheal enterotoxins, 0.06 to 0.36 ng/ml in *satchu* for *Salmonella*, and 0.08 ng/ml in *khyopeh* to 0.38 ng/ml in beef *kargyong* for staphylococcal enterotoxins (Table 6). Additionally, from cultural isolates, molecular detection of a few enterotoxin genes was also performed by the PCR technique using strain-specific primers. *S. piscifermentans* BSLST44 and *S. piscifermentans* BULST54 isolated from beef *kargyong* and *S. aureus* PSST53 isolated from pork *kargyong* were found positive for the *sea* virulent gene (Figure 5A). *B. cereus* BSMB16 isolated from beef *kargyong* was found negative for *nhea* and *nheb* genes (Figure 5B), and *S. enterica* SMX21 isolated from *satchu* was also tested negative for *stn* and *invA* virulence genes (Figure 5B).

**TABLE 5:** Detection of enterotoxins in traditionally processed meat extracts by enzyme-linked immunosorbent assay.

Sample tested	Collection site	Results with the Tecra kit		Results with the Ridascreen set kit
		*Bacillus* diarrheal enterotoxins	*Salmonella* enterotoxins	Staphylococcal enterotoxins
Beef *kargyong* (*n* = 2)	Martam, East Sikkim	+	−	+
*Satchu* (*n* = 2)		+	+	−
Beef *kargyong* (*n* = 2)	Lalbazaar, Gangtok	−	−	−
Pork *kargyong* (*n* = 2)		−	−	+
*Satchu* (*n* = 2)		−	−	−
Beef *kargyong* (*n* = 2)	Kitam, South Sikkim	−	−	−
Pork *kargyong* (*n* = 2)		−	−	−
Beef *kargyong* (*n* = 2)	Geyzing, West Sikkim	−	−	−
Pork *kargyong* (*n* = 2)		−	−	−
*Satchu* (*n* = 2)		−	−	−
Pork *kargyong* (*n* = 2)	Burtuk, Gangtok	−	+	−
Yak *kargyong* (*n* = 2)	Lachung, North Sikkim	−	−	−
*Khyopeh* (*n* = 2)		−	−	−
Positive control		+	+	+
Negative control		−	−	−

**n = number of sample; results were consistent in duplicate tests with each of the enterotoxins in the tested samples*.*

**TABLE 6:** Minimum amount of enterotoxins in sample extracts detectable by Tecra and RIDASCREEN kits.

Sample tested^a^	Collection site	Minimum detectable amount (ng/ml)^b^ of enterotoxins		
		Bacillus diarrheal enterotoxins	*Salmonella* enterotoxins	Staphylococcal enterotoxins
Beef *kargyong* (*n* = 2)	Martam, East Sikkim	0.35	0.12	0.38
		0.11	0.09	0.17
*Satchu* (*n* = 2)		0.31	0.36	0.19
		0.28	0.31	0.12
Beef *kargyong* (*n* = 2)	Lalbazaar, Gangtok	0.15	0.16	0.15
		0.13	0.19	0.10
Pork *kargyong* (*n* = 2)		0.16	0.18	0.31
		0.09	0.14	0.12
*Satchu* (*n* = 2)		0.13	0.16	0.16
		0.11	0.08	0.11
Beef *kargyong* (*n* = 2)	Kitam, South Sikkim	0.13	0.08	0.16
		0.08	0.17	0.09
Pork *kargyong* (*n* = 2)		0.16	0.15	0.13
		0.18	0.11	0.18
Beef *kargyong* (*n* = 2)	Geyzing, West Sikkim	0.09	0.10	0.09
		0.10	0.15	0.18
Pork *kargyong* (*n* = 2)		0.14	0.13	0.13
		0.11	0.17	0.11
*Satchu* (*n* = 2)		0.12	0.12	0.10
		0.15	0.15	0.16
Pork *kargyong* (*n* = 2)	Burtuk, Gangtok	0.18	0.34	0.12
		0.16	0.13	0.15
Yak *kargyong* (*n* = 2)	Lachung, North Sikkim	0.08	0.18	0.18
		0.12	0.13	0.16
*Khyopeh* (*n* = 2)		0.16	0.15	0.13
		0.18	0.06	0.08
Positive control		2.81	2.74	3.52
Negative control		0.08	0.12	0.11

**^*a*^Sample extracts were prepared by using the phosphate-buffered saline described above. ^*b*^The values are averages for duplicate assays*.*

### Prevalence of Antimicrobial Resistant Bacteria

**FIGURE 6:**
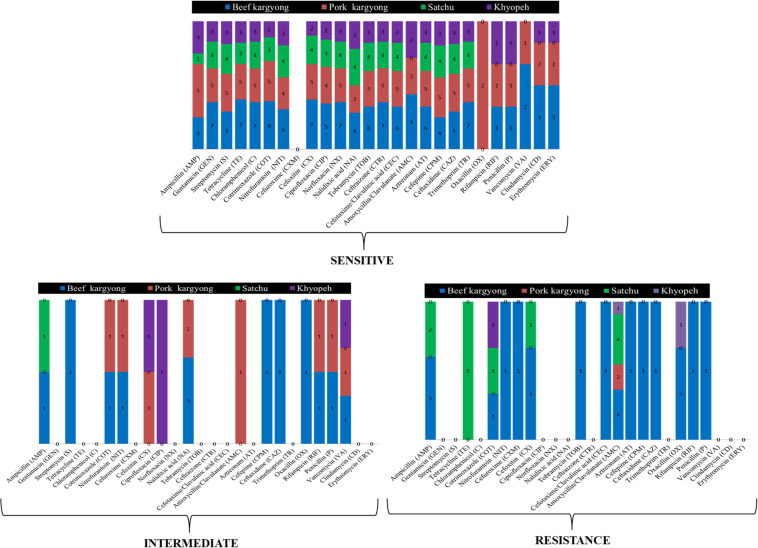
Bacterial isolates from different meat samples showing variable patterns of being sensitive, intermediate, and resistant against the tested antibiotics.

Bacteria isolated from traditionally processed meat products showed 100% susceptibility against gentamicin, cotrimoxazole, norfloxacin, and trimethoprim. However, a variable resistance pattern was also observed (Supplementary Tables 1–3). Overall, the highest level of resistance was observed in amoxicillin–clavulanate (58%) followed by ampicillin (27%). All Gram-positive bacteria were found sensitive against clindamycin and erythromycin, but a resistance pattern was observed in oxacillin (50%) followed by penicillin (33%) and ampicillin (27%) (Supplementary Tables 3, 4). Nitrofurantoin was found resistant to *K. pneumoniae* (BSE27) isolated from beef *kargyong*. Cotrimoxazole was found resistant to *E. faecalis* KHE57 isolated from *khyopeh*. The bacterial isolates from different meat samples showed variable patterns of being sensitive, intermediate, and resistant toward various classes of antibiotics which are shown in Figure 6.

## Discussion

In the present study, we collected four types of traditionally processed meat products (beef *kargyong*, pork *kargyong*, *satchu*, and *khyopeh*) from different places of Sikkim, and they were analyzed for pH, moisture, microbial load, spoilage/pathogenic bacteria, enterotoxins, and antibiotic susceptibility test. The pH value of the samples varied from 5.3 to 5.9 which was similar to the pH value found in traditional dry-cured lacon (5.0–6.0) (Lorenzo et al., 2015), beef and pork jerky (5.5–6.07) (Yang et al., 2018), and biltong (5.0–6.2) (Petit et al., 2014). Preservation of meat by drying has been practiced for centuries and the traditional meat products of Sikkim are preserved either by sun drying or smoking. Among the tested samples, we observed a very less moisture content in *khyopeh* with 2.5 to 18% in beef *kargyong*. The quality and stability of meat products are greatly affected by pH and moisture content (Frazier and Westhoff, 2014). Phenotypic characterization of the 128 bacterial isolates was performed for presumptive identification followed by molecular characterization of representative strains (19) targeting the 16S rRNA gene. The identified bacteria include *S. piscifermentans*, *C. freundii*, *E. faecalis*, *S. enterica*, *S. aureus*, *C. werkmanii*, *K. pneumoniae*, *M. caseolyticus*, *K. aerogenes*, *S. saprophyticus*, *P. anthropi*, *C. europaeus*, *S. sonnei*, *E. fergusonii*, *K. grimontii*, *B. cepacia*, and *B. cereus*. *S. aureus* is usually considered as a food-poisoning bacterium (Hennekinne et al., 2012), but it is frequently underreported since the illness caused by it is mild (Paparella et al., 2018). Foodborne illness caused by *S. aureus* is often associated with the consumption of meat and meat products (Madahi et al., 2014; Hasanpour Dehkordi et al., 2017). The prevalence of *S. aureus* in meat and dairy products has been reported earlier (Guven et al., 2010; Momtaz et al., 2013; Hasanpour Dehkordi et al., 2017; Rahi et al., 2020). Coagulase-negative staphylococci (CoNS) like *S. saprophyticus* and *S. piscifermentans* have been reported as flavoring microorganisms capable of reducing nitrite, enhancing color stability, and preventing rancidity by inhibiting the oxidation of unsaturated free fatty acids (Talon and Leroy, 2006). Many dried/salted/fermented meat products are often reported to be dominated by coagulase-negative staphylococci (Lorenzo et al., 2015), which may contribute to bioprotection against foodborne pathogens by producing bacteriocins (Mainar et al., 2017). We found *sea* enterotoxin gene from isolated strains of *S. aureus* and a few CoNS. Similar studies were reported in ground beef where enterotoxin and biofilm genes in coagulase-negative *Staphylococcus* were detected, indicating it as a potential pathogen (Pavan et al., 2019). Although *Staphylococcus* produces different types of enterotoxins like the classical enterotoxins (sea–see) and the newer enterotoxins (seg–sely) (Fisher et al., 2018), however, strains producing sea are implicated in the majority of cases of staphylococcal food poisoning (Kérouanton et al., 2007; Wallin-Carlquist et al., 2010). The presence of *sea* virulent gene in a few samples of *kargyong* advocates the concern for food safety of the product, which might have been contaminated during traditional processing; however, there has been no official report of food-poisoning cases in Sikkim after the consumption of *kargyong*.

We also identified various bacteria belonging to the family Enterobacteriaceae, which are commonly present in the intestines of human (Martinson et al., 2019) and animal (Schierack et al., 2007) that may survive in diverse environments (Martinson et al., 2019). However, they may also cause a variety of community-acquired (foodborne) and nosocomial infections (Bereket et al., 2012). *Klebsiella* has often been reported in meat products as a contaminant from water, containers, and feces of animal or human origin (Gundogan et al., 2013). Foods are commonly contaminated with *Salmonella* and *Shigella* by an infected food handler who practices poor hygiene and inadequate cooking (Garedew et al., 2016; Liu et al., 2018; Ranjbar et al., 2020). *Salmonella* is one of the most common bacterial pathogens in laboratory-confirmed foodborne illness cases causing gastroenteritis, typhoid fever, and bacteremia (Eng et al., 2015). The major identified virulence genes like *inv*, *stn*, *fimA*, and *spv* responsible for salmonellosis are linked to a combination of plasmid and chromosomal factors (Chaudhary et al., 2015). Dry meat products are traditionally considered as safe food; however, the outbreak of foodborne illness caused by *S. enterica* is often reported due to its ability to withstand the adverse environment leading to cross-protection against abiotic stress (Mutz et al., 2020). Although the ELISA test detected *Salmonella* spp. in a few tested samples, the PCR method showed the absence of *invA* and *stn* virulence genes in the isolated strain. The *invA* gene is primarily targeted for the identification of *Salmonella* in food of animal origin, since it is present in pathogenic serovars and has been demonstrated to be a rapid and effective method for diagnosis of salmonellosis (Karmi, 2013; Carraturo et al., 2016). However, there are few reports on the absence of *invA* gene in certain *Salmonella* strains, which indicate that they are not invasive or may have other invasive mechanisms (Kaushik et al., 2014; Sharma and Das, 2016; Buehler et al., 2019; Kadry et al., 2019; Yanestria et al., 2019).

Enterococci isolated from meat products have the ability to produce enterocins harboring antimicrobial activity against *L. monocytogenes* (da Costa et al., 2019). *B. cereus* is an endospore-forming bacterium responsible for foodborne illness in humans and is frequently involved in foodborne outbreaks (Tewari et al., 2015). *Bacillus* enterotoxins have been reported from fermented legume products like *soumbala* and *bikalga* of Africa (Ouoba et al., 2008); doenjang, a fermented soybean food of Korea (Lee et al., 2017); dairy milk (Saleh-Lakha et al., 2017); some street food of Indian Himalayas (Kharel et al., 2016); and meat products (Soleimani et al., 2018). *B. cepacia* is identified as a spoilage microorganism in food (Cunningham-Oakes et al., 2019).

The widespread use of antibiotics in animals for the treatment of diseases and other purposes may develop antibiotic resistance in bacteria resulting into cross-transmission of resistance genes from animals to humans through food (Safarpoor Dehkordi et al., 2017; Tang et al., 2017). The antibiogram of bacterial isolates showed a maximum rate of sensitivity against gentamicin and trimethoprim, which is in accordance with a similar report where 90% of sensitivity was observed in bacterial isolates from street foods (Amor et al., 2019). The majority of isolated strains showed the highest resistance pattern against amoxicillin–clavulanate followed by ampicillin. Amoxicillin–clavulanate-resistant bacteria were also reported from retail sausage in Malaysia which exhibited 100% resistance along with penicillin followed by ampicillin (83.3%) and cefotaxime (71.4%) (Tew et al., 2016). The increasing prevalence of amoxicillin–clavulanate resistance has been reported due to the continued spread of β-lactamase-mediated resistance (White et al., 2004). We also found a strain of bacteria identified as *K. pneumoniae* that showed resistance against nitrofurantoin which is a broad-spectrum antibiotic used for the treatment of uncomplicated urinary tract infections (UTI) (Osei Sekyere, 2018). Most of the Gram-positive cocci were oxacillin resistant; however, the resistance pattern was not observed in any cephalosporin class of antibiotics except for *B. cereus*. Overall, the majority of the isolated strains from traditionally processed meat products of Sikkim showed a maximum percentage of sensitivity against the tested antibiotics.

## Conclusion

The traditional processing of perishable meat into flavor-enhanced products by drying, smoking, and fermentation is culturally and organoleptically accepted by ethnic people of Sikkim in India. This study showed the prevalence of some spoilage and pathogenic bacteria; however, enterotoxin virulent genes were not detected in bacterial strains isolated from traditional meat products except for the *sea* virulent gene which tested positive from two samples of *kargyong*. It was interesting to find out the absence of pathogenic bacteria and enterotoxins in *khyopeh*, which is a fermented yak meat product found only in the north district of Sikkim. Most of the bacterial strains in traditionally processed meat products of Sikkim showed a maximum percentage of sensitivity against the tested antibiotics. Some samples of meat products showed the prevalence of spoilage bacteria probably as contaminants during the traditional processing method. Hence, proper monitoring of hygienic conditions during preparation practices is highly recommended for the safety of these culturally acceptable meat products.

## Data Availability Statement

The datasets generated for this study can be found in online repositories. The names of the repository/repositories and accession number(s) can be found in the article/Supplementary Material.

## Author Contributions

NT and JT: conceptualization, resources, supervision, and writing—review and editing. MB: data curation, formal analysis, methodology, software, visualization, and roles/writing—original draft. JT: funding acquisition and project administration. MB and NT: investigation. MB, NT, and JT: validation. All authors contributed to the article and approved the submitted version.

## Conflict of Interest

The authors declare that the research was conducted in the absence of any commercial or financial relationships that could be construed as a potential conflict of interest.

## Footnotes

1 http://technelysium.com.au/ChromasPro.html
