# Supplementary material for: Molecular Characterization of Bacteria, Detection of Enterotoxin Genes, and Screening of Antibiotic Susceptibility Patterns in Traditionally Processed Meat Products of Sikkim, India
Source: Front Microbiol. 2021 Jan 11;11:599606. doi: 10.3389/fmicb.2020.599606 (PMC7830132; doi:10.3389/fmicb.2020.599606)
Supplement: Supplementary file 1 [file Data_Sheet_1.docx]

| **Table 1: Antibiotic susceptibility test of Gram-negative bacteria isolated from meat products of Sikkim** | | | | | | | | | | | | | | | | | | |
| --- | --- | --- | --- | --- | --- | --- | --- | --- | --- | --- | --- | --- | --- | --- | --- | --- | --- | --- |
| **Antimicrobial agents** | **Disk content**  **(mcg)** | **Interpretative Criteria as per CLSI guidelines (mm)** | | | **Isolates with Zone of Inhibition (mm)** | | | | | | | | | | | | | |
|  |  |  |  |  | **BSE32** | | **SME36** | | **PSE39** | | **BSE17** | | **BSE41** | | **SME33** | | **PSKE30** | |
|  |  | **S** | **I** | **R** |  |  |  |  |  |  |  |  |  |  |  |  |  |  |
| **Ampicillin (AMP)** | 10 | ≥17 | 14-16 | ≤13 | 18 | S | 14 | R | 18 | S | 15 | I | 13 | R | 16 | I | 22 | S |
| **Gentamicin (GEN)** | 10 | ≥15 | 13-14 | ≤12 | 18 | S | 18 | S | 17 | S | 17 | S | 16 | S | 17 | S | 21 | S |
| **Streptomycin (S)** | 10 | ≥15 | 12-14 | ≤11 | 14 | I | 17 | S | 16 | S | 16 | S | 14 | I | 16 | S | 20 | S |
| **Tetracycline (TE)** | 30 | ≥15 | 12-14 | ≤11 | 16 | S | 21 | S | 18 | S | 19 | S | 19 | S | 19 | S | 21 | S |
| **Chloramphenicol (C)** | 30 | ≥18 | 13-17 | ≤12 | 23 | S | 24 | S | 20 | S | 21 | S | 22 | S | 22 | S | 25 | S |
| **Cotrimoxazole (COT)** | 25 | ≥16 | 11-15 | ≤10 | 24 | S | 23 | S | 23 | S | 22 | S | 24 | S | 24 | S | 22 | S |
| **Nitrofurantoin (NIT)** | 300 | ≥17 | 15-16 | ≤14 | 18 | S | 19 | S | 16 | S | 16 | I | 18 | S | 17 | S | 16 | I |
| **Cefuroxime (CXM)** | 30 | ≥18 | 15-17 | ≤14 | 18 | S | 22 | S | 19 | S | 19 | S | 20 | S | 22 | S | 21 | S |
| **Cefoxitin (CX)** | 30 | ≥18 | 15-17 | ≤14 | 19 | S | 20 | S | 17 | I | NZ | R | NZ | R | NZ | R | 23 | S |
| **Ciprofloxacin (CIP)** | 5 | ≥21 | 16-20 | ≤15 | 27 | S | 27 | S | 25 | S | 25 | S | 25 | S | 30 | S | 25 | S |
| **Norfloxacin (NX)** | 10 | ≥17 | 13-16 | ≤12 | 27 | S | 24 | S | 26 | S | 26 | S | 23 | S | 29 | S | 27 | S |
| **Nalidixic acid (NA)** | 30 | ≥19 | 14-18 | ≤13 | 24 | S | 20 | S | 18 | I | 16 | I | 17 | I | 20 | S | 16 | I |
| **Tobramycin (TOB)** | 10 | ≥15 | 13-14 | ≤12 | 15 | S | 16 | S | 16 | S | 16 | S | 15 | S | 16 | S | 21 | S |
| **Ceftraizone (CTR)** | 30 | ≥23 | 20-22 | ≤19 | 25 | S | 25 | S | 25 | S | 24 | S | 26 | S | 27 | S | 26 | S |
| **Cefotaxime/Clavulinic acid (CEC)** | 30 | ≥18 | 15-17 | ≤14 | 26 | S | 24 | S | 26 | S | 24 | S | 28 | S | 28 | S | 32 | S |
| **Amoxycillin/Clavulanate (AMC)** | 30 | ≥18 | 14-17 | ≤13 | NZ | R | NZ | R | NZ | R | NZ | R | NZ | R | NZ | R | 14 | I |
| **Aztreonam (AT)** | 30 | ≥21 | 18-20 | ≤17 | 24 | S | 26 | S | 23 | S | 21 | S | 27 | S | 27 | S | 33 | S |
| **Cefepime (CPM)** | 30 | ≥25 | 19-24 | ≤18 | 25 | S | 28 | S | 25 | S | 22 | I | 25 | S | 30 | S | 25 | S |
| **Ceftaxidime (CAZ)** | 30 | ≥21 | 18-20 | ≤17 | 23 | S | 23 | S | 22 | S | 20 | I | 22 | S | 25 | S | 23 | S |
| **Trimethoprim (TR)** | 5 | ≥16 | 11-15 | ≤10 | 20 | S | 18 | S | 20 | S | 19 | S | 18 | S | 17 | S | 20 | S |

CLSI=Clinical and Laboratory Standards Institute, NZ= No zone, S= sensitive, I= intermediate, R= resistance

| Table 2: Antibiotic susceptibility test of Gram-negative bacteria isolated from meat products of Sikkim | | | | | | | | | | | | | | | | |
| --- | --- | --- | --- | --- | --- | --- | --- | --- | --- | --- | --- | --- | --- | --- | --- | --- |
| Antimicrobial agents | **Disk content**  **(mcg)** | **Interpretative Criteria as per CLSI guidelines (mm)** | | | **Isolates with Zone of Inhibition (mm)** | | | | | | | | | | | |
|  |  |  |  |  | **SME26** | | **BSE27** | | **SMX21** | | **KHE40** | | **KHE57** | | **PSE31** | |
|  |  | **S** | **I** | **R** |  |  |  |  |  |  |  |  |  |  |  |  |
| Ampicillin (AMP) | 10 | ≥17 | 14-16 | ≤13 | 17 | S | 10 | R | 5 | R | 22 | S | 26 | S | 19 | S |
| Gentamicin (GEN) | 10 | ≥15 | 13-14 | ≤12 | 16 | S | 18 | S | 17 | S | 17 | S | 16 | S | 18 | S |
| Streptomycin (S) | 10 | ≥15 | 12-14 | ≤11 | 16 | S | 17 | S | 15 | S | 19 | S | 16 | S | 17 | S |
| Tetracycline (TE) | 30 | ≥15 | 12-14 | ≤11 | 16 | S | 18 | S | NZ | R | 22 | S | 26 | S | 21 | S |
| Chloramphenicol (C) | 30 | ≥18 | 13-17 | ≤12 | 19 | S | 23 | S | 22 | S | 26 | S | 21 | S | 24 | S |
| Cotrimoxazole (COT) | 25 | ≥16 | 11-15 | ≤10 | 21 | S | 20 | S | 20 | S | 25 | S | NZ | R | 23 | S |
| Nitrofurantoin (NIT) | 300 | ≥17 | 15-16 | ≤14 | 18 | S | 12 | R | 17 | S | 19 | S | 18 | S | 19 | S |
| Cefuroxime (CXM) | 30 | ≥18 | 15-17 | ≤14 | 20 | S | 19 | S | 21 | S | 23 | S | 25 | S | 22 | S |
| Cefoxitin (CX) | 30 | ≥18 | 15-17 | ≤14 | 19 | S | 18 | S | 18 | S | 22 | S | 17 | I | 20 | S |
| Ciprofloxacin (CIP) | 5 | ≥21 | 16-20 | ≤15 | 25 | S | 22 | S | 29 | S | 30 | S | 16 | I | 27 | S |
| Norfloxacin (NX) | 10 | ≥17 | 13-16 | ≤12 | 24 | S | 22 | S | 28 | S | 26 | S | 17 | S | 24 | S |
| Nalidixic acid (NA) | 30 | ≥19 | 14-18 | ≤13 | 21 | S | 18 | I | 22 | S | 23 | S | 22 | S | 20 | S |
| Tobramycin (TOB) | 10 | ≥15 | 13-14 | ≤12 | 17 | S | 16 | S | 17 | S | 17 | S | 16 | S | 16 | S |
| Ceftraizone (CTR) | 30 | ≥23 | 20-22 | ≤19 | 24 | S | 16 | S | 28 | S | 28 | S | 26 | S | 25 | S |
| Cefotaxime/Clavulinic  acid (CEC) | 30 | ≥18 | 15-17 | ≤14 | 27 | S | 24 | S | 25 | S | 24 | S | 25 | S | 24 | S |
| Amoxycillin/Clavulanate (AMC) | 30 | ≥18 | 14-17 | ≤13 | NZ | R | NZ | R | NZ | R | NZ | R | 19 | S | NZ | R |
| Aztreonam (AT) | 30 | ≥21 | 18-20 | ≤17 | 22 | S | 29 | S | 22 | S | 24 | S | 23 | S | 26 | S |
| Cefepime (CPM) | 30 | ≥25 | 19-24 | ≤18 | 26 | S | 22 | I | 25 | S | 30 | S | 26 | S | 28 | S |
| Ceftaxidime (CAZ) | 30 | ≥21 | 18-20 | ≤17 | 23 | S | 21 | S | 26 | S | 26 | S | 23 | S | 23 | S |
| Trimethoprim (TR) | 5 | ≥16 | 11-15 | ≤10 | 21 | S | 23 | S | 26 | S | 19 | S | 24 | S | 15 | S |

CLSI=Clinical and Laboratory Standards Institute, NZ= No zone, S= sensitive, I= intermediate, R= resistance

| Table 3: Antibiotic susceptibility test of Gram-positive bacteria isolated from meat products of Sikkim | | | | | | | | | | | | | | | | |
| --- | --- | --- | --- | --- | --- | --- | --- | --- | --- | --- | --- | --- | --- | --- | --- | --- |
| Antimicrobial agents | **Disk content**  **(mcg)** | **Interpretative Criteria as per CLSI guidelines (mm)** | | | **Isolates with Zone of Inhibition (mm)** | | | | | | | | | | | |
|  |  |  |  |  | **KHST43** | | **BULST54** | | **PSST49** | | **PSST53** | | **BSLST44** | | **BSMB16** | |
|  |  | **S** | **I** | **R** |  |  |  |  |  |  |  |  |  |  |  |  |
| Ampicillin (AMP) | 10 | ≥17 | 14-16 | ≤13 | 32 | S | 27 | S | 25 | S | 18 | S | 17 | S | 11 | R |
| Gentamicin (GEN) | 10 | ≥15 | 13-14 | ≤12 | 20 | S | 20 | S | 26 | S | 15 | S | 16 | S | 22 | S |
| Streptomycin (S) | 10 | ≥15 | 12-14 | ≤11 | 22 | S | 17 | S | 18 | S | 17 | S | 15 | S | 20 | S |
| Tetracycline (TE) | 30 | ≥15 | 12-14 | ≤11 | 26 | S | 21 | S | 18 | S | 17 | S | 18 | S | 23 | S |
| Chloramphenicol (C) | 30 | ≥18 | 13-17 | ≤12 | 22 | S | 20 | S | 19 | S | 21 | S | 20 | S | 19 | S |
| Cotrimoxazole (COT) | 25 | ≥16 | 11-15 | ≤10 | 24 | S | 23 | S | 27 | S | 18 | S | 21 | S | 10 | R |
| Nitrofurantoin (NIT) | 300 | ≥17 | 15-16 | ≤14 | 18 | S | 19 | S | 24 | S | 22 | S | 21 | S | 20 | S |
| Cefuroxime (CXM) | 30 | ≥18 | 15-17 | ≤14 | 27 | S | 21 | S | 25 | S | 20 | S | 28 | S | 12 | R |
| Cefoxitin (CX) | 30 | ≥18 | 15-17 | ≤14 | 22 | S | 26 | S | 24 | S | 19 | S | 22 | S | 20 | S |
| Ciprofloxacin (CIP) | 5 | ≥21 | 16-20 | ≤15 | 28 | S | 25 | S | 26 | S | 22 | S | 23 | S | 24 | S |
| Norfloxacin (NX) | 10 | ≥17 | 13-16 | ≤12 | 26 | S | 23 | S | 27 | S | 25 | S | 23 | S | 28 | S |
| Nalidixic acid (NA) | 30 | ≥19 | 14-18 | ≤13 | 20 | S | 20 | S | 19 | S | 21 | S | 24 | S | 19 | S |
| Tobramycin (TOB) | 10 | ≥15 | 13-14 | ≤12 | 15 | S | 16 | S | 17 | S | 25 | S | 16 | S | 12 | R |
| Ceftraizone (CTR) | 30 | ≥23 | 20-22 | ≤19 | 24 | S | 24 | S | 23 | S | 25 | S | 26 | S | 25 | S |
| Cefotaxime/Clavulinic  acid (CEC) | 30 | ≥18 | 15-17 | ≤14 | 26 | S | 24 | S | 23 | S | 24 | S | 25 | S | 16 | R |
| Amoxycillin/Clavulanate (AMC) | 30 | ≥18 | 14-17 | ≤13 | 22 | S | 20 | S | 22 | S | 21 | S | 20 | S | 19 | S |

CLSI=Clinical and Laboratory Standards Institute, NZ= No zone, S= sensitive, I= intermediate, R= resistance

| Table 4: Antibiotic susceptibility test of Gram-positive bacteria isolated from meat products of Sikkim | | | | | | | | | | | | | | | | |
| --- | --- | --- | --- | --- | --- | --- | --- | --- | --- | --- | --- | --- | --- | --- | --- | --- |
| **Antimicrobial agents** | **Disk content**  **(mcg)** | **Interpretative Criteria as per CLSI guidelines (mm)** | | | **Isolates with Zone of Inhibition (mm)** | | | | | | | | | | | |
|  |  |  |  |  | **KHST43** | | **BULST54** | | **PSST49** | | **PSST53** | | **BSLST44** | | **BSMB16** | |
|  |  | **S** | **I** | **R** |  |  |  |  |  |  |  |  |  |  |  |  |
| Aztreonam (AT) | 30 | ≥21 | 18-20 | ≤17 | 23 | S | 22 | S | 23 | S | 21 | S | 23 | S | 15 | R |
| Cefepime (CPM) | 30 | ≥25 | 19-24 | ≤18 | 26 | S | 27 | S | 28 | S | 27 | S | 26 | S | 11 | R |
| Ceftaxidime (CAZ) | 30 | ≥21 | 18-20 | ≤17 | 22 | S | 23 | S | 21 | S | 22 | S | 23 | S | 16 | R |
| Trimethoprim (TR) | 5 | ≥16 | 11-15 | ≤10 | 23 | S | 21 | S | 24 | S | 21 | S | 23 | S | 25 | S |
| Oxacillin (OX) | 1 | ≥18 | - | ≤17 | 14 | R | 14 | R | 23 | S | 19 | S | 14 | I | 10 | R |
| Rifampicin (RIF) | 5 | ≥26 | 23-25 | ≤23 | 26 | S | 24 | I | 25 | I | 26 | S | 27 | S | 15 | R |
| Penicillin (P) | 10 | 29 | - | 28 | 29 | S | 28 | I | 24 | R | 29 | S | 32 | S | NZ | R |
| Vancomycin (VA) | 30 | ≥17 | 15-16 | ≤14 | 16 | I | 15 | I | 15 | I | 18 | S | 18 | S | 18 | S |
| Clindamycin (CD) | 2 | ≥21 | 15-20 | ≤14 | 24 | S | 22 | S | 21 | S | 24 | S | 23 | S | 21 | S |
| Erythromycin (ERY) | 15 | ≥23 | 14-22 | ≤13 | 24 | S | 25 | S | 24 | S | 23 | S | 24 | S | 25 | S |

CLSI=Clinical and Laboratory Standards Institute, NZ= No zone, S= sensitive, I= intermediate, R= resistance
